# Pharmacokinetic/Pharmacodynamic Determinations of Iron-tannic Molecular Nanoparticles with its Implication in MR Imaging and Enhancement of Liver Clearance

**DOI:** 10.7150/ntno.63310

**Published:** 2022-01-01

**Authors:** Thipjutha Phatruengdet, Piyachat Khuemjun, Jannarong Intakhad, Saowalak Krunchanuchat, Arpamas Chariyakornkul, Rawiwan Wongpoomchai, Chalermchai Pilapong

**Affiliations:** 1 Center of Excellence for Molecular Imaging (CEMI), Department of Radiologic Technology, Faculty of Associated Medical Sciences, Chiang Mai University, Chiang Mai 50200, Thailand.; 2 Department of Biochemistry, Faculty of Medicine, Chiang Mai University, Chiang Mai, 50200, Thailand.

**Keywords:** MRI agent, liver imaging, liver clearance, autophagy, efferocytosis

## Abstract

Assessment and enhancement of liver clearance are promising strategies for protection of liver from various liver diseases. Iron-tannic nanoparticles (FTs) were previously considered as imageable autophagic enhancers with biodegradation potential. Herein, we present a new approach for utilizing Iron-tannic nanoparticles (FTs) as a tool for imaging and increasing liver clearance. Pharmacokinetic profiling suggested that FTs were initially found in blood circulation and thereafter were distributed to the liver. By using MR imaging (T_1_ weighted), maximum MRI signal enhancement was found to occur after 30 minutes post-injection (i.v.) and gradually decreased afterward. Decreasing MRI signal may be due to FTs metabolism by the liver. By assessing imaging-derived pharmacokinetics, we can simply determine the rate constant of liver degradation of FTs. Potentially, we might use this parameter to monitor liver function, where its clearance is of concern. Once functional implication of FTs in liver clearance was investigated, FTs were found to induce hepatocyte autophagy along with activation of lysosomes. Consequently, the hepatocytes were capable of efficiently clearing cellular debris. From these results, it is clear that FTs should be considered as a molecular tool for quantitative MRI-derived liver function assessment, and for enhancing clearance function in liver parenchyma. Hopefully, our findings will pave the way to develop new strategies for non-invasive assessment and enhancement of liver clearance.

## Introduction

It is well documented that the liver plays a crucial role in various biological processes such as metabolism and detoxification. The liver is known to be an RES system that is essential for liver clearance homeostasis such as detoxification of toxins, clearance of cell debris, etc. 1. Generally, multiple cell types such as immune cells, epithelial cells, and hepatocytes are thought to be involved in liver clearance homeostasis. Both innate immune cells (e.g. macrophages, monocyte, and dendritic cells) and adaptive immune cells such as lymphocytes work together for liver clearance via phagocytosis and lysosomal degradation 2-4. In addition, hepatocytes also play a role in liver clearance function, but the exact mechanism is not well understood 5. Presently, liver diseases such as cirrhosis, hepatitis, and HCC, accounts for approximately 2 million deaths per year worldwide. It is expected that more than 75 million people are at risk of alcohol-associated liver disease and over 2 billion are at risk of non-alcoholic fatty liver disease and hepatocellular carcinoma 6-8. Notably, early stages for such liver disease are associated with malfunctional liver clearance function. Therefore, non-invasive assessment of liver clearance function is a promising approach for early detection and monitoring of various kinds of liver disease 9.

Magnetic resonance imaging (MRI) is powerful and non-invasive imaging technique that can provide three-dimensional image with a high resolution. Because of its limited sensitivity, MRI contrast agents have been developed especially for Gadolinium-based contrast agents (GdCAs) 10. However, various commercially available GdCAs have been suspended due to their adverse effect to nephrogenic system and renal function 11. Nowadays, Gd^3+^-free MRI CAs (e.g. Fe-based and Mn-based) have been developed as safe alternative 12-14. In last decade, plant-polyphenol based nanoparticles have been extensively developed as theranostic agents 15-17. Among them, iron-polyphenol nanoparticles are particularly of interest in both disease diagnosis (by MRI) and treatment because they can integrate imaging moieties and therapeutic agents within the same platform 13,14,18-21. Different platforms of iron-polyphenol nanoparticles exhibited unique properties including physio-chemical properties, MRI relaxation properties as well as biological properties (Physio-chemical properties and MRI relaxation properties of different iron-polyphenol nanoparticles were comparatively provided in Table S1). Previously, we introduced nanoparticles of iron-tannic complexes (so-called FTs), which are considered as a new class of magnetic resonance imaging (MRI) agents for *in vivo* imaging 13. The FTs exhibit ideal properties for biomedical applications due to their small size with narrow distribution, acceptable MRI relaxivity (r_1_ = 3.14 s^-1^mM^-1^), and unique stability with biodegradable potential 14. Unlike conventional solid nanomaterials, the FTs are considered to be molecular nanoparticles assembled from iron-tannic complexes though non-covalent interaction; they behave like molecular matter rather than crystalline solids such as metal and semiconductor nanoparticles. As a result, assembling structure of FTs can be changed or are degradable in the response to specific milieu. For example, an acidic environment is able to change the coordination state of FTs from Tris-dominant to be Bis-dominant structures 22; reactive oxygen species like H_2_O_2_ are able to cleave Fe-O bonds of FTs. In addition, due to the existence of catechol group and metal-ligand bonding in FTs 23, covalent and coordinate interaction between FTs and biomolecules can occur in living subjects 15. Moreover, tannic itself is also metabolized by specific enzymes and chemicals in the liver 24,25. This suggested that FTs provide ideal characteristics for being a biodegradable material. Previously, we showed that FTs were capable of accumulating within the liver of Wistar rats and enhance the MRI signals 13. As far as FTs degradation and MRI signal are concerned, the capability of enhancing MRI signal in T_1_-weigthed technique decreases as FTs degradation occurs. If FTs degradation is mediated by liver clearance mechanism, we would expect a diminishing of MRI signal. Therefore, measurement of these processes could become a parameter for assessment of liver clearance function.

Autophagy and related functions are important for liver clearance, as dysfunction of autophagy has been implicated in the initiation and progression of various liver diseases. Autophagy defects lead to impairment in the recycling biomolecules and clearance of cellular debris 26-28. In NAFLD, insulin resistance, excess lipid accumulation, endoplasmic reticulum (ER), and oxidative stress are able to induce impairment of autophagy in hepatocytes 29,30. Defective autophagy also plays a role in pathogenesis of HCC by enhancing inflammation, oxidative stress, and other issues 31-33. In addition, hepatic autophagy is an essential mechanism for liver regeneration, and impaired autophagy is found to be related to dysfunction of various processes of hepatic regeneration. These facts justify the development of potential pharmacological strategies for inducing autophagy as a promising mean for protecting the liver from initiation and progression of liver diseases. Currently, a large number of studies are devoted to restoring the autophagic activity for various liver diseases, and they have achieved partial success 34-36. In our previous works, we found that FTs can induce autophagy in various cell lines including hepatocytes without serious toxicity *in vitro* and *in vivo* 13,14,37. In fact, FTs are non-mutagenic and non-carcinogenic, have low acute toxicity, and no sub-acute toxicity in Wistar rats (not shown here). Therefore, apart from being utilized in liver clearance assessment, FTs may also be a potential pharmacological method for autophagy enhancement.

In this research, we aimed to investigate *in vivo* pharmacokinetics of FTs by MRI and measured liver iron accumulation in order to determine the parameters to be utilized for assessment of liver clearance function. In addition, the capabilities of inducing autophagy of FTs along with functional implication for cell death clearance were also checked.

## Methods

### FTs preparation

The FTs were synthesized according to our previous report 13. Typically, the FT was simply obtained by mixing ferric chloride (Sigma-Aldrich) and tannic acid (Loba Chemie) in a PBS buffer (pH 7.4) at room temperature for a few minutes in ambient air. The obtained FTs were purified through dialysis and were further concentrated by heating at 90 °C. The concentrated FTs were repeatedly purified by dialysis and were further filtered through a 0.22 μm syringe filter cellulose acetate membrane for sterilization. The concentration of the FTs was determined and expressed as FTs concentration (for *in vivo*) and having an equivalent concentration of iron (for *in vitro*).

### Animal used protocol

Male Wistar rats were purchased from Nomura Siam International Co., Ltd. Bangkok, Thailand. The rats (6 week) were divided into different groups according to injection times. The rats were intravenously injected with FTs 5.5 mg/kg bw. The rats were further maintained for different lengths of time in a controlled environment with a 12-h/12-h light/dark cycle and provided with food and water ad libitum. It should be noted that the dose for injection is 5.5 mg/kg bw which was determined from our toxicology study. This animal use protocol was approved by the Animal Ethics Committee of Faculty of Medicine, Chiang Mai University, Thailand (Protocol Number: 09/2562).

### Magnetic resonance imaging (MRI) and analysis

At the desired time points after injection, the rats were anaesthetized using 40 mg/kg bw of Thiopental (NAARI). After that, abdominal MRI scanning of rats was performed on a Philips Achieva 1.5T MRI scanner at ambient temperature (25 °C) using turbo spin echo with the following imaging parameters; repetition time 450 ms; echo time 8 ms; slide thickness 3.0 mm. After scanning, the MRI images were selected to determine region of interest (ROI) and signal intensity (SI) of liver, kidneys, and bladder was measured using Philip DICOM viewer R3.0software. The SI of each organs was compared to that measured on the muscle of each plane, (reported as corrected SI).

### Iron determination with spectrophotometer

Non-protein bound iron from whole liver was determined by this method. As typically done, weighted liver samples were homogineoused using a doune homogenizer. The homoginates were then centrifuged at 15000 rpm at 4 °C for 30 minutes. Afterward, the supernatants were collected and mixed with TCA:HCl (1:1) solution. The solutions were heated at 95 °C for 1 hour. After cooling down to room temperature, the solutions were centrifuged at 14500 rpm for 30 minutes. Then, 200 μL of the supernatants were collected and mixed with 50 μL HNO_3_ (69%) for 30 minutes. Finally, the iron content in such solutions was quantified by mean of UV-Vis spectrophotometer (Agilent 8453) using thiocyanate assay, as described in previous work. The iron content determined by this method was reported as milligrams of iron per milligrams of protein (measured by Bradford assay).

### Total iron determination by ICP-OES

Total iron content in whole liver was determined by this method. Weighted liver samples were homogenised in 1:1 HCl (37%):HNO_3_ (69%) at room temperature overnight. The homogenates were spun down at 10000g for 10 minutes. Afterward, the supernatants were collected and were further mixed with 2% HNO_3_. Next, the iron content in such solutions was quantified by ICP-OES (Perkin Elmer) according to standard protocol using different concentrations of FeCl_3_ as standard calibration solutions. The iron content determined by this method was reported as microgram of iron per gram of liver wet weight.

### Prussian blue staining

Prussian blue staining of formalin-fixed and paraffin-embedded (FFPE) liver sections (3 μm) was performed according to our previous report. After deparaffinization and rehydration, the sections were stained in a mixture of 5% HCl and 5% potassium ferrocyanide (Merck) for 30 minutes at room temperature, and subsequently were co-stained with 0.2% neural red (May & Baker) for 5 minutes. After that, the stained sections were observed under microscope (Nikon, Eclipse Ts2).

### Immunofluorescence staining of LC3B in liver sections

After deparaffinization and rehydration of FFPE liver sections according to a standard protocol, antigen retrieval was conducted by boiling in 10 mM sodium citrate buffer. After cooling down, the slides were permaebilized and blocked using 0.4% Triton X-100 (BioBasic) and 5% BSA (Capricorn) in PBS, consecutively. After that, the slides were incubated with an anti-LC3B antibody (Thermo Fisher Scientific) overnight at 4 °C. After twice washing with PBS (10 minutes per wash), the slides were further incubated with a Dylight 488 conjugated anti-rabbit secondary antibody (Thermo Fisher Scientific) for 2 hours in the dark at room temperature. Finally, the slides were washed and mounted by using Permount (Merck) for further analysis under fluorescence microscope.

### Western blotting analysis of LC3B/p62 in liver tissue

The liver tissues were homogenized in RIPA buffer (BioBasic) containing proteinase inhibitor cocktail (Nacalai) and spun down for 30 minutes at 12,000 rpm, 4 °C. The protein concentration of the supernatant was determined by Bradford assay. Then, standard gel electrophoresis was performed, and the proteins were transferred onto PVDF membrane (Millipore). Next, immunoblotting was performed with OmniPAGE Mini**-**PAGE blotting set (Cleaver scientific). Primary antibodies including GAPDH (Affinity), SQSTM/p62 (Affinity), MAP1LC3B (Elabscience), and HRP-conjugated rabbit anti-rat IgG (Boster) were used. Chromogenic detection was performed using DAB Chormogenic kit (Boster).

### Biodegradation of FTs

The FTs solution was incubated with esterase (Sigma) and hydrogen peroxide for different lengths of time, the degradation of FTs was then monitored by measuring the metal-to-ligand charge transfer band (MLCT) using UV-Vis spectrophotometer (Agilent 8453).

### Leakage of free iron

Certain amount of FTs were added into dialysis bag (12 kDa MWCO) and the sealed dialysis bag was further placed in a 50 mL tube. The FTs solution was dialyzed against PBS for different lengths of time. The dialysate was collected at desired time of dialysation for iron quantification by KSCN assay. The leakage of free iron from FTs was calculated as percentage of iron leakage.

### Cell culture

Mouse hepatocyte cell line (Alpha mouse liver 12, AML12) was purchased from ATCC and cultured in DMEM HAM F-12 (Caisson Laboratories) supplemented with 10% Fetal bovine serum (Hyclone), 1% prenicilin-streptomycin (Caisson Laboratories) at 37 °C with 5% CO_2_.

### Intracellular iron accumulation

The cells were seeded in 24 well plates, pre-coated with collagen type I (US biological) for 24 hours. Then, the cells were treated with FTs (100 μM) for 1 hour at 37 °C with/without NaN_3_, and at 4 °C. After that, the cells were trypsinized, and were further lyzed with a mixed acid solution (1:1 of HCl: HNO_3_) at 60 °C for 30 minutes. Next, the lysed solutions were subjected for iron quantification by the KSCN assay.

### TEM analysis

The cells were seeded in 6 well plates (pre-coated with collagen type I) for 24 hours. Then, the cells were treated with FTs (100 μM) for 24 hours**.** After washing, standard procedures for TEM sample preparation were performed such as fixation, dehydration, infiltration, embedding, polymerisation, and sectioning. Finally, the thin sections were observed under the TEM (JEM-2200FS) with an accelerating voltage of 200 kV.

### Live cell fluorescent imaging

The cells being treated in different experimental conditions were stained with different fluorescence probes for different purposes. For example; Hoechst33342 (ApexBio)/Propidium iodide (US Biological) for cell death determination; and Lysotracker Red DND99 (Thermo Fisher Scientific) for lysosome staining. In addition, cell debris tracking was also carried out. At first, cellular debris was prepared by maintaining cells over a logarithmic phase of growth until only cell debris were obtained. After washing, the cell debris was labeled with fluorescein isothiocyanate (Sigma) by incubating overnight at 4 °C. After washing, cell debris was collected for conducting cell debris tracking by fluorescent imaging.

## Results and Discussion

**Figure 1:**
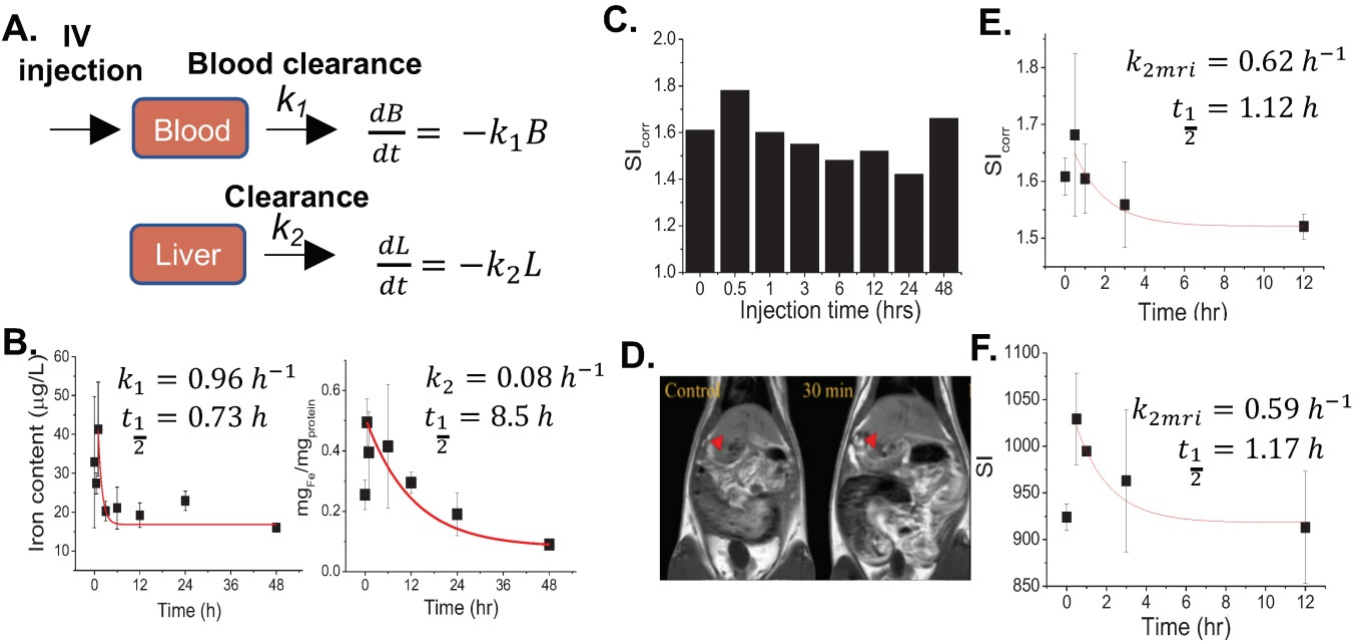
** (a)** Pharmacokinetic model of FTs clearance. Blood clearance and liver clearance of FTs are represented by *k*_1_ and *k*_2_, respectively. **(b)** Representative plots of serum (left) and liver iron content (right) versus injection time with corresponding fitted curves, as well as rates of clearance and half-life (n=2). **(c)** Representative signal intensity (SI_corr_) time courses in liver of rats after injection with FTs. **(d)** Typical abdominal T_1_ weighted images of control rats (without FTs injection) and rats injected with FTs for 30 minutes (0.5 hrs). **(e,f)** representative plots of liver SI time courses with corresponding fitted curves, as well as rates of liver clearance and half-life (n=2).

According to our previous report regarding toxicology tests in healthy rats, it was found that LD 50 value of FTs was higher than 55 mg.kg^-1^ b.w. Therefore, herein, we chose a dose that was 10 times lower than LD50 (5.5 mg.kg^-1^ b.w.) for investigating pharmacokinetic and pharmacodynamic profiles 13. At first, we hypothesized that FTs would be cleared from blood circulation, then they are distributed to the liver. We then determined iron content in serum and the liver after intravenous injection of FTs for a different time. It was found that iron content in serum decayed rapidly initially, and those in the liver also reached a plateau at around 30 minutes and decreased thereafter (Figure 1b), suggesting that the FTs can be rapidly cleared from blood circulation, and then can be distributed to the liver and eliminated from the liver later on. From this data, we were able to estimate pharmacokinetic parameters using simplified first-order decay (Figure 1a). Blood clearance rate constant and half-life were calculated as 0.96 hr^-1^and 0.73 hr (Figure 1b, left), respectively, indicating their excellent *in vivo* pharmacokinetic properties for imaging examinations in organ of interest. In the liver, we parameterized the pharmacokinetic profile of FTs regarding their elimination by considering data occurring after plateau (Figure 1b, right). By doing so, we were able to estimate liver decay rate constant and half-life of FTs as 0.08 hr^-1^ and 8.5 hr (Figure. 1b, right). By evaluating the results from MRI analysis, a similar trend was observed in a comparison with iron content in the liver, at which maximum MRI enhancement was obtained at 30 minutes post-injection (i.v.) and gradually decreases thereafter (Figure 1c) (typical abdominal MRI images are shown in Figure 1d). Similarly, we fitted MRI data during period time of FTs elimination (after peak to 12 hours) using a simplified first-order decay, and the decay rate constant and half-life were determined as 0.62 hr^-1^ and 1.12 hours, respectively, for corrected SI analysis, which is quite consistent with those determined by using un-corrected SI (Figure 1e,f). Because such pharmacokinetic parameters are involved in liver elimination processes, we might take advantage of this to monitor liver function, whenever liver clearance functions are of concern. In addition to the liver, FTs were also actually able to distribute to other organs such as the kidneys and bladder. As the results show in Figure S1, the MRI signal from kidneys and bladder plateau at around 30-60 minutes. This indicates that the FTs are also able to be distributed to the kidneys and were cleared through the kidneys to the bladder. These pharmacokinetic data is comparable to GdCA and MnCA, which were found to targeting the liver and clearing via predominant hepatic pathways as well as renal system 12.

**Figure 2:**
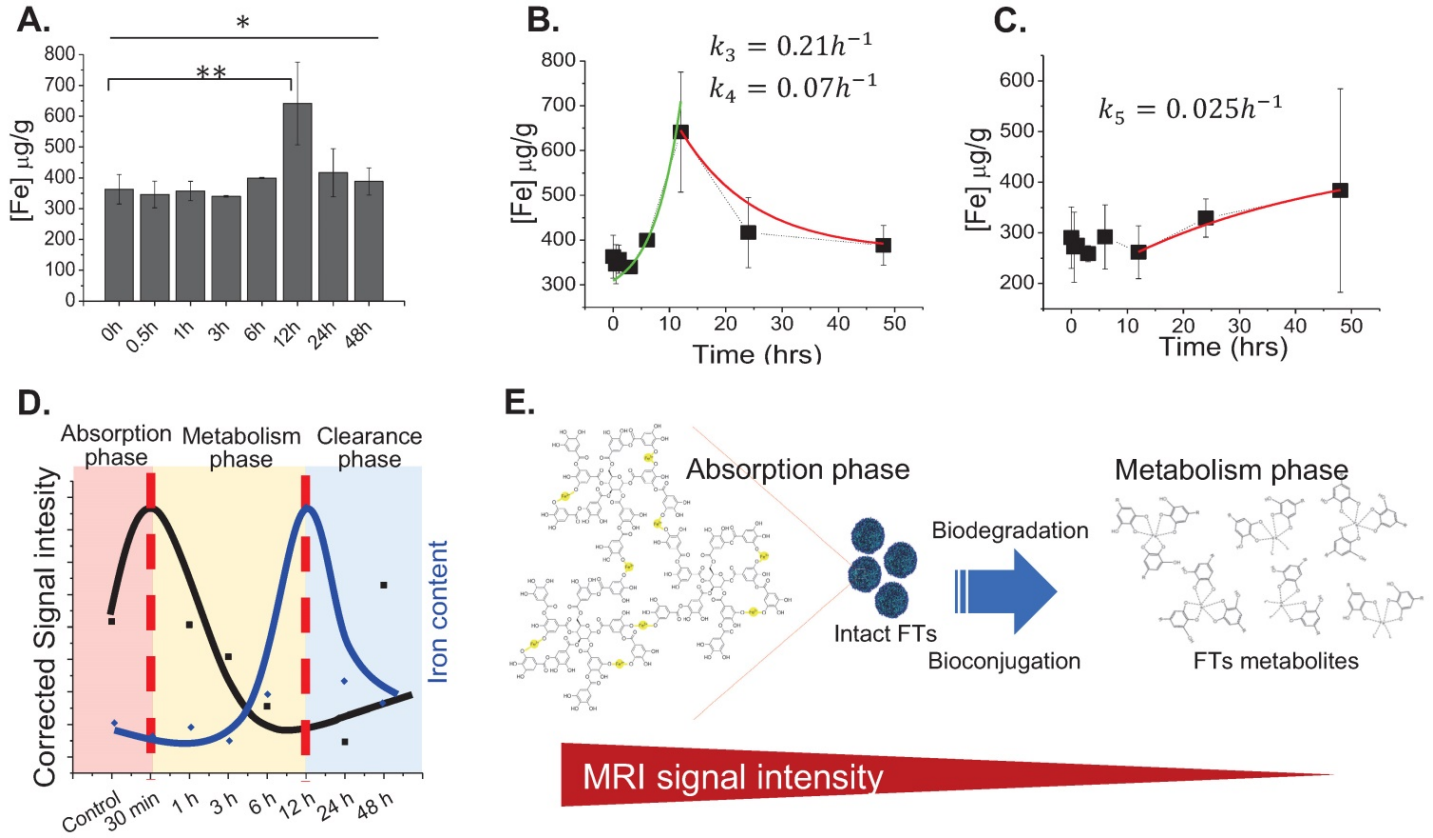
** (a)** Total iron content in liver of rats after being injected with FTs for different lengths of time. *P<0.05, **P<0.01 (n=2). **(b)** Corresponding fitted curves and liver metabolism rate of FTs (*k*_3_) and clearance rate of FTs metabolites (*k*_4_) (n=2). **(c)** Representative plot of kidney iron content versus time of injection with corresponding fitted curves, as well as rates of kidney absorption (*k*_5_) (n=2). **(d)** Correlation between liver MRI signal and total iron content determination with predicted pharmacokinetic parameters including FTs absorption, FTs metabolism, and clearance. **(e)** Schematic illustration of structural and chemical changes of FTs undergoing biodegradation and bioconjugation in liver with predicted MRI signal intensity during such processes.

From the above results, we hypothesized that FTs could be metabolized by the liver. Next, we tried to determine the total amount of iron content per liver weight (without protein precipitation) in order to check the amount of iron in all forms, except blood. Surprisingly, the iron content was found to increase with time and peaked at 12 hours and decreased thereafter (Figure 2a), which is different from the observations listed above (Figure 1b), where the iron content had reached plateau at around 30 minutes. It should be noted that the amount of iron measured without protein precipitation represents total iron content including FTs and iron-bound proteins. However, iron measured after protein precipitation represents the amount of FTs, as well as non-protein bound iron. An increase of total iron content during the decreasing of FTs might be due to FTs metabolism, where FTs metabolites preferentially accumulate in the liver. After 12 hours of injection, the iron content was found to decrease, indicating that the FTs metabolites can somehow be removed from the liver. By fitting the data using simplified one-compartment model, we were able to independently parameterize the rate constants of FTs metabolism (*k*_3_) and FTs metabolites clearance (*k*_4_) in liver as 0.21hr^-1^ and 0.07hr^-1^, respectively (Figure 2b). In the kidneys, the amount of iron was found to increase as the time increases, especially after 12 hours of injection or during liver clearance phase, implying that metabolized FTs are able to distribute to kidneys for renal clearance with the rate constant of kidney absorption (*k*_5_) being at 0.025 hr^-1^ (Figure 2c). According to maximum value of iron accumulation in liver and kidney, compared to base line level, the iron accumulation in liver was found to be 300 µg over baseline, but that of kidney was 100 ug. Therefore, this can be inferred that that hepatobiliary pathway is dominant for FTs clearance (3 times).

**Figure 3:**
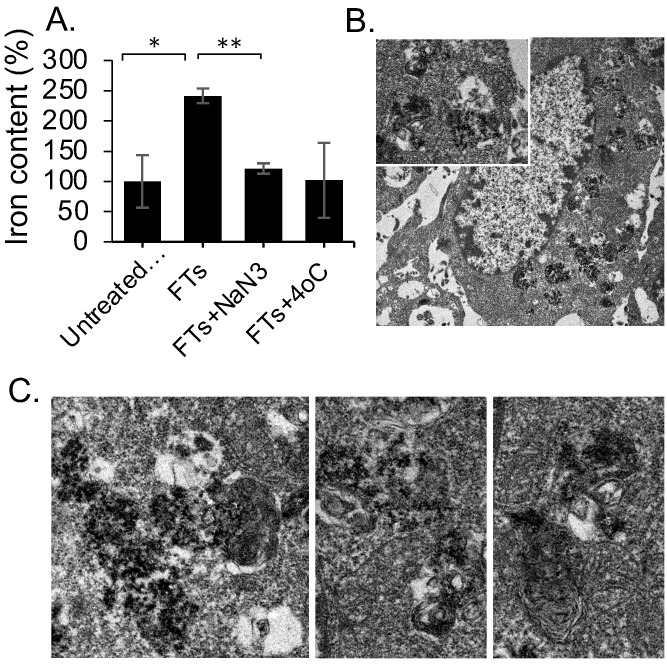
** (a)** Change in iron content in AML12 cells after incubation with FTs and FTs with ATP-depleted conditions (NaN_3_ and 4°C), *P<0.05, **P<0.01 (n =3) **(b)** representative TEM images of AML12 cell with the presence of FTs in vesicular structures. **(c)** TEM images of ultrastructure of digestive machineries containing FTs with different levels of assembly and degradation.

From the above-mentioned results regarding MR imaging and total iron content determination, we summarized the pharmacokinetic profiles of FTs in the liver that is shown in Figure 2d. At initial time of injection, FTs are able to accumulate in the liver, during what is called the absorption phase. In this phase, FTs are presented as an intact form, whereby no degradation occurs. As time passes, the FTs might undergo metabolic phase, involving breakdown and bioconjugation reactions, where unassembled iron complexes are possibly presented (as shown in Figure 2e). Even though, FTs were quite stable toward trans-chelation and trans-metalation 14, as well as iron leakage in PBS buffer (Figure S2), as is known, TA can be hydrolyzed by hydrolases, releasing glucose and one or more smaller phenolic acid. Similar to TA, possibly, the structure of the assembled iron-tannic complexes in FTs can be broken down into a smaller size by the hydrolysis 38. In order to monitor the hydrolysis mediated FTs degradation, liver porcine esterase was used as hydrolase representative and time-dependent FTs degradation mediated by esterase was performed. As shown in Figure S3, the absorbance of charge transfer (CT) band of FTs 14, were found to decrease as incubation time with esterase increases, implying that esterase plays a role as a metabolic mechanism of FTs in the liver. In addition to enzyme mediated FTs degradation, H_2_O_2_, which is usually found in the liver plays a role in the chemical modification of several amino acids and proteins 39-41, and it also plays a role in FTs breakdown. As the results show in Figure S4a, absorbance of charge transfer (CT) band of FTs is found to decrease when incubated with H_2_O_2_ in both a concentration and time-dependent manner. Because absorbance of CT band is directly related to the number of ferric-catechol, decreases in CT band absorption indicates the breakdown of FTs by cleaving coordinate bonds between ferric-catechol in FTs, thereby becoming a faded color of FTs solution (Figure S4b), which is in good accordance with previous report 23. In this metabolic phase, it is expected that degraded FTs diminish their MRI signal enhancement efficiency due to their smaller size 42. To confirm this tendency, we performed T_1_ weighted MRI scanning of a solution containing degraded and non-degraded FTs. It is clearly seen that the MRI signal of degraded FTs is dramatically decreased, compared to the non-degraded ones (Figure S4c). As such, we can imply that decreasing MRI signal after peak is attributed to degradation of FTs. Subsequently, bioconjugations of FTs complexes could occur by their interaction with other biomolecules via bio-transchelation and nucleophilic addition of TA 15, leading to high accumulation of iron at 12 hours, which is likely to be protein-bound iron complexes. Finally, the metabolized FTs are cleared from the liver and secreted thru the renal system. However, the exact mechanism for FTs metabolism is not yet clear. Furthermore, it was noticed from Prussian blue staining that accumulation of FTs was found in liver parenchyma (Figure S5), where 80% of the cell is hepatocytes, suggesting that FTs are taken up by liver hepatocytes. In an attempt to understand how hepatocytes take up and metabolize FTs, *in vitro* experiments using murine hepatocytes cell line were conducted. We hypothesized that the FTs were taken up by hepatocytes via endocytosis mechanisms. At first, we determined intracellular accumulation of FTs by measuring the iron content within the cells. Apparently, the FTs treated cells show a higher amount of iron content, compared to untreated cells. However, as expected, incubation of the cells with FTs in the presence of ATP-depleted conditions shows a decrease in the iron content, compared to FTs (Figure 3a). Furthermore, we attempted to observe the presence of FTs in acidic organelles such as endosomes, lysosomes, etc., which is considered as a crucial cellular physiology of endocytosis 43, and a possible sink of FTs metabolism 9,44. As observed in TEM imaging of the cells treated with FTs, it is clearly seen that the FTs are mainly found in endosomal vesicles (Figure 3b). These results suggest that the FTs can be taken up by the cells via endocytosis pathways. The presence of FTs in the endosomal vesicles might be critical for their metabolism at which various digestive enzymes and chemicals are found (including hydrolase, H_2_O_2_ and others). Notably, we further observed endolysosomes and fusion of endosomes and lysosomes, containing different levels of FTs assembly and degradation (Figure 3c), suggesting that FTs can be targeted for degradation in such machineries. In addition, the presence of FTs in acidic organelles might be vital for their bioconjugation via transchelation because the Bis-dominant coordination state presented in an acidic condition is easily trans-chelated by endogenous ligands, in comparison with Tris-dominant coordination state of intact FTs 22.

**Figure 4:**
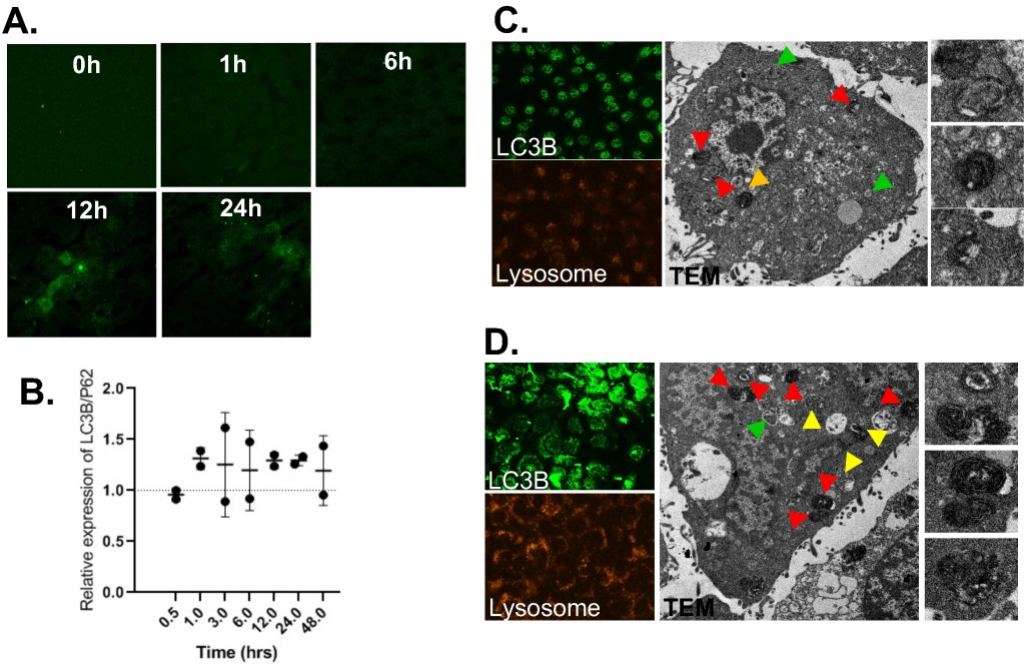
** (a)** LC3B immunofluorescence staining of liver sections of rats injected with FTs for different times. **(b)** Western blotting analysis of relative changes in LC3B expression with respect to p62 expression, reported as LC3B/p62, in liver tissues of rats injected with FTs for different times. **(c,d)** LC3B staining, Lysotracker staining and TEM analysis of autophagy associated vesicles of (c) untreated cells and ((d) FTs-treated cells. Green arrows indicate autophagosome, red and yellow arrows indicate autolysosome with different digestive stages. Right panel shows snap-shot images of representative lysosome fusion for liver clearance.

Next, we investigated liver pharmacodynamics of FTs, implicated in having protection potential. Previously, we indicated that FTs were capable of inducing autophagy and cellular clearance function in various cell lines, including hepatocytes. Herein, we determined an expression of autophagic markers in liver sections 45. As observed in immunofluorescence staining of LC3B (Figure 4a), expression of LC3B is found to increase as injection time increases, especially in hepatocytes, consistent with FTs accumulation in liver parenchyma. However, increase in LC3B is solely not a criterion in enhanced autophagy. Determination of autophagy flux would be more valuable for measurement of autophagy activity. As is known, p62 or sequestosome 1 (SQSTM1) is widely used as an autophagy marker and is degraded by autophagy 46,47. Detection of LC3B together with p62 (LC3B/p62) can be used to monitor autophagy activity and only when LC3B has increased and P62 has decreased, thus enhanced autophagic could be concluded 48. The quantitative level of LC3B/p62 was detected by Western Blotting. As seen in Figure 4b, enhanced relative LC3B/p62 expression is observed in FTs injected rats, compared to control rats, indicating that liver autophagy is activated by FTs.

**Figure 5:**
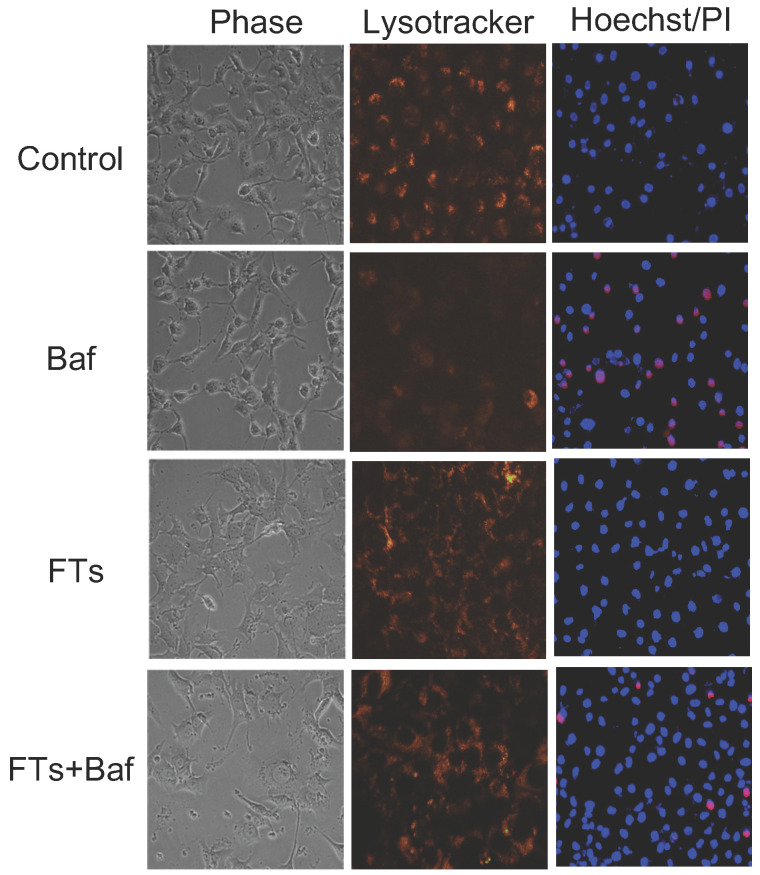
Phase contrast images, fluorescence images of lysotracker staining and fluorescence images Hoechst/PI staining of untreated cells (control), Baf-treated cells, FTs-treated cells and the cells co-treated with FTs and Baf for 48 hours. Final concentrations of FTs and Baf were 100 μM and 50 nM, respectively

Although liver macrophage plays a role in liver clearance functions, hepatocytes are also found to play a role in this, as well 5. Therefore, we proposed functional implication of FTs-mediated liver clearance and explored this in the hepatocyte cell lines. At first, we induced autophagy and activated lysosome in the cells using FTs. As expected, FTs are capable of inducing LC3B expression and lysosomal activation along with the formation of autophagosomes and autolysosome 49 (Figure 4c,d), confirming that FTs play a role in induction of liver autophagy and clearance. It is well known that lysosome plays a crucial role in cellular degradation and autophagy 50. We showed that inhibition of lysosome acidification by Bafilomycin A (Baf) resulted in decreasing of lysosome signal and induced cell death (Figure 5 and Figure S5). Obviously, the residual cells from Baf treatment exhibit dramatic changes in cell morphology and lose their attachment efficiency. It can be implied that the cells with lysosome dysfunction are prone to lose their biophysical features and even cell death can result. Interestingly, FTs are able to recover lysosome and biophysical dysfunctions, and protect against cell death induced by Baf (Figure 5).

In order to show the functional implications of FTs-mediated liver clearance, we treated the cells with cellular debris (FITC labeled) and checked its accumulation together with lysosome signal. It can be seen that cellular debris is clearly found to accumulate in the control cells. A higher accumulation of the debris is observed in Baf-treated cells, suggesting that inhibition of lysosome acidification reduces clearance activity of the cells. Interestingly, less accumulation of cellular debris is observed in FTs-treated cells, demonstrating functional clearance of hepatocytes had been induced by FTs. In the presence of Baf, FTs are also able to restore the clearance function of the cells, as observed by a lessened signal of cellular debris. Consistent with these findings, lysosome is found to highly activate along with FTs-mediated clearance, especially in the presence of Baf, confirming functional implication of FTs in hepatocyte clearance. However, there is little known regarding the molecular mechanisms of debris clearance mediated by FTs.

## Conclusion

FTs were found to exhibit both diagnosis and protection potential for the liver. The former potential takes advantage of their degradability and pharmacokinetic data including MRI, in which decreasing MRI signal is associated with liver clearance function. Therefore, we can utilize FTs as a molecular imaging tool for noninvasively monitoring liver function in the context of its clearance. The latter potential stems from capability of FTs in activation of biological processes and molecular functions associated with liver clearances such as autophagy, lysosomal degradation, and cell debris clearance. This will allow us to develop a new iron supplement for prevention and therapy of various liver diseases.

## Supplementary Material

Supplementary figures and tables.

## Figures and Tables

**Figure 6:**
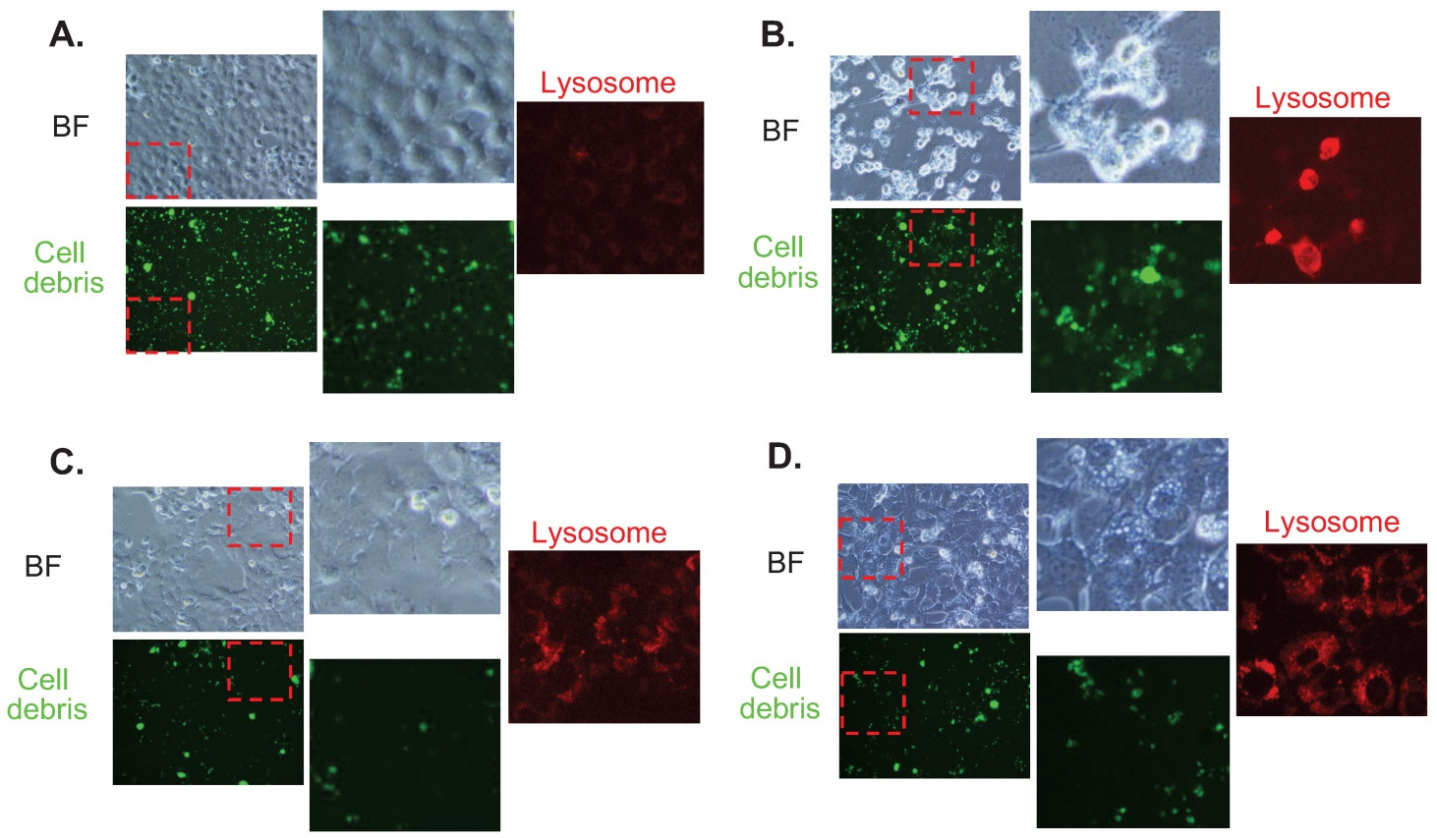
Fluorescence imaging for cell debris tracking and lysosome of **(a)** untreated cells (control), **(b)** Baf-treated cells, **(c)** FTs-treated cells, and **(d)** the cells co-treated with FTs and Baf for 48 hours
