# Supplementary material for: Pharmacokinetic/Pharmacodynamic Determinations of Iron-tannic Molecular Nanoparticles with its Implication in MR Imaging and Enhancement of Liver Clearance
Source: Nanotheranostics. 2022 Jan 1;6(2):195–204. doi: 10.7150/ntno.63310 (PMC8671955; doi:10.7150/ntno.63310)
Supplement: Supplementary file 1 — Supplementary figures and tables. [file ntnov06p0195s1.pdf]

Supporting Information

Pharmacokinetic/Pharmacodynamic Determinations of Iron-tannic Molecular Nanoparticles with its Implication in MR Imaging and Enhancement of Liver Clearance

Thipjutha Phatruengdet, [a] Piyachat Khuemjun, [a] Jannarong Intakhad, [a] Saowalak Krunchanuchat, [a] Arpamas Chariyakornkul, [b] Rawiwan Wongpoomchai, [b] and Chalermchai Pilapong *[a]

[a]Center of Excellence for Molecular Imaging (CEMI), Department of Radiologic Technology, Faculty of Associated Medical Sciences, Chiang Mai University, Chiang Mai 50200, Thailand

[b]Department of Biochemistry, Faculty of Medicine, Chiang Mai University, Chiang Mai, 50200, Thailand

*Corresponding Email: chalermchai.pilapong@cmu.ac.th

Table S1. Some properties of iron-polyphenol nanoparticles

Platforms	Physical size (nm)	Hydrodynamic size (nm)	r_1 $(mM^{-1}s^{-1})$	r_2 $(mM^{-1}s^{-1})$	reference
FeIII-TA capsule	3000	NA	2.04	12	(1)
Fe-CPNDs	1.5	2	1.5	2.9	(2)
Fe-EA	200	240	0.36	61.1	(3)
Fe-GA-PEG	5	20	3.5	0.97	(4)
FTs (this work)	3	8	3.14	NA	(5, 6)

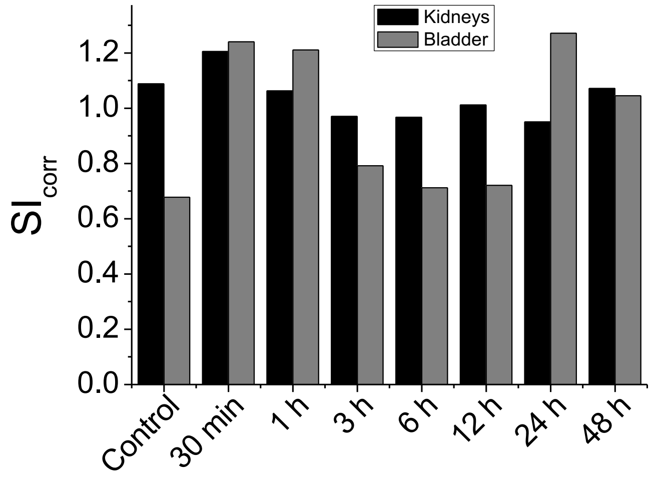

Figure S1 corrected MRI signal intensity in kidneys and bladder of rate being intravenously injected for different lengths of time

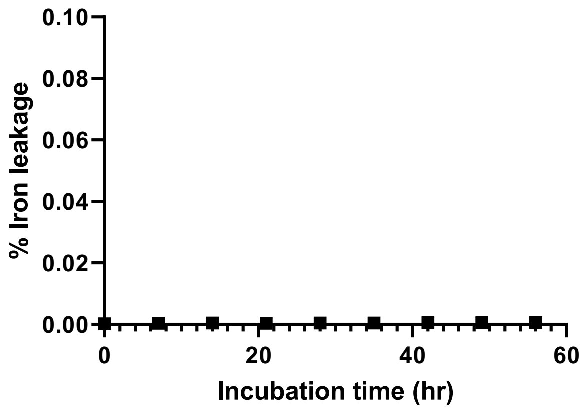

Figure S2 Percentage of iron leakage from FTs

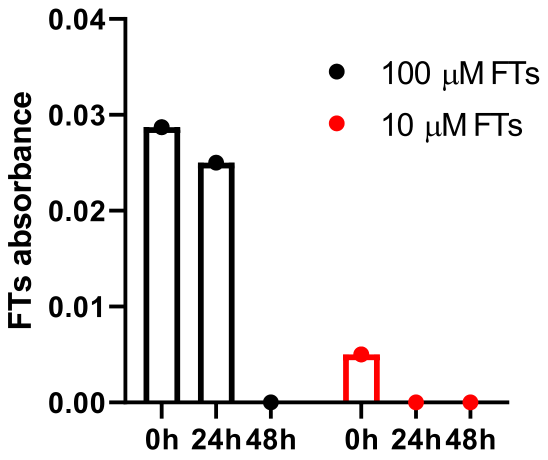

Figure S3 Absorbance of FTs incubated with esterase (1mg) for different length of time.

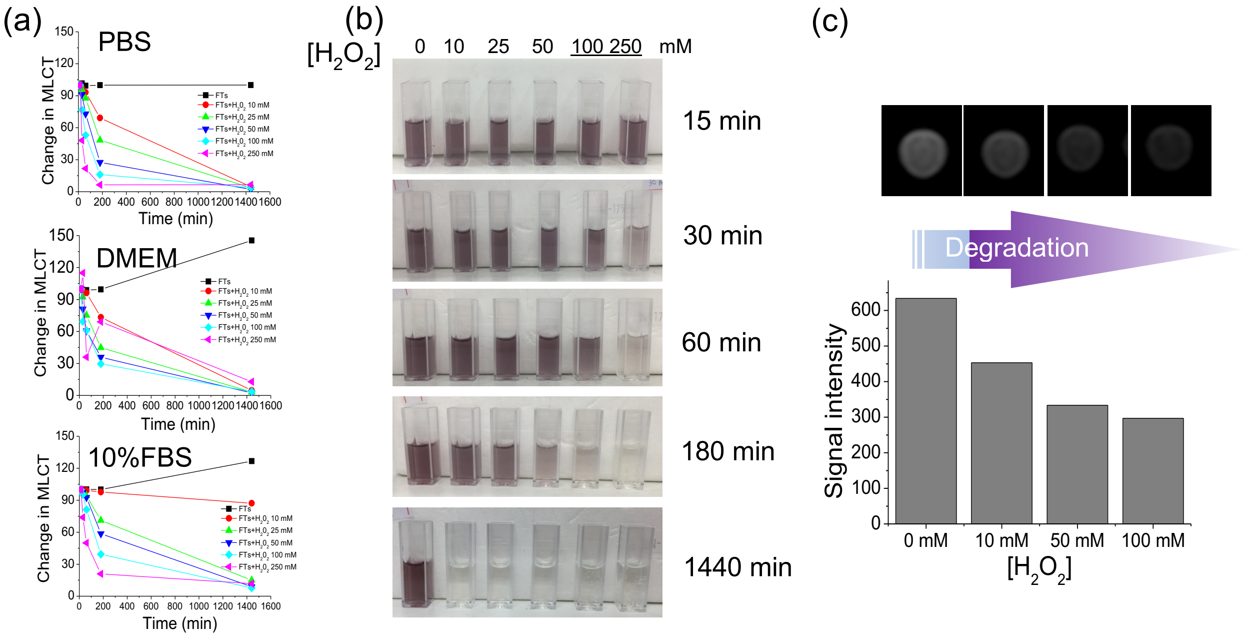

Figure S4 (a) Changes in absorbance of CT band of FTs incubated with different concentrations of H_2O_2 for different lengths of incubation time with different media (b) Photographic images of the FTs solution in PBS containing H_2O_2. (c) T_1 weighted images with corresponding signal intensity of FTs solutions with different degrees of degradation (different H_2O_2 concentrations).

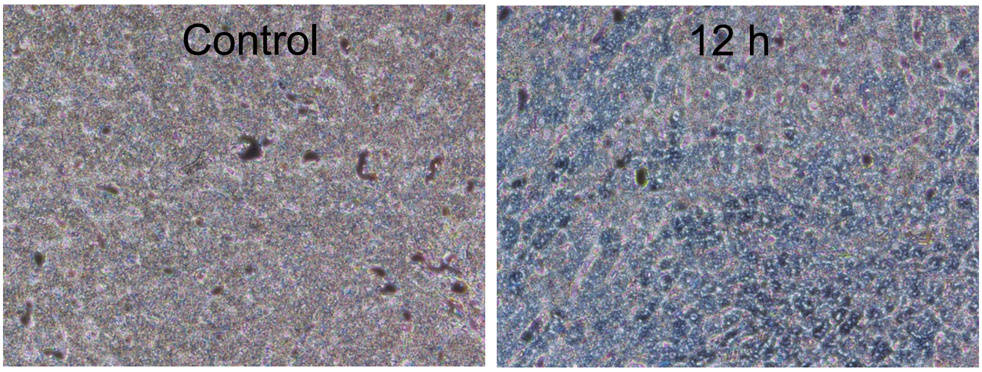

Figure S5 Prussian blue staining of liver section from control rats and rats injected with FTs for 12 hours

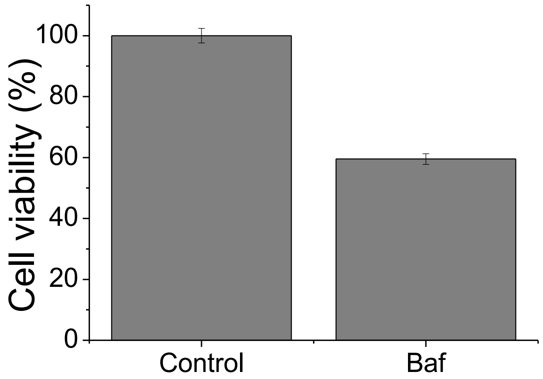

Figure S6 MTT assay of AML12 cells after treatment with Baf (50 nM) for 48 hours.

References

1. Guo J, Ping Y, Ejima H, Alt K, Meissner M, Richardson JJ, et al. Engineering multifunctional capsules through the assembly of metal-phenolic networks. Angewandte Chemie (International ed in English). 2014;53(22):5546-51.
2. Liu F, He X, Chen H, Zhang J, Zhang H, Wang Z. Gram-scale synthesis of coordination polymer nanodots with renal clearance properties for cancer theranostic applications. Nature communications. 2015;6:8003.
3. Zhao G, Wu H, Feng R, Wang D, Xu P, Jiang P, et al. Novel Metal Polyphenol Framework for MR Imaging-Guided Photothermal Therapy. ACS applied materials & interfaces. 2018;10(4):3295-304.
4. Jin Q, Zhu W, Jiang D, Zhang R, Kutyreff CJ, Engle JW, et al. Ultra-small iron-gallic acid coordination polymer nanoparticles for chelator-free labeling of (64)Cu and multimodal imaging-guided photothermal therapy. Nanoscale. 2017;9(34):12609-17.
5. Phatruengdet T, Intakhad J, Tapunya M, Chariyakornkul A, Hlaing CB, Wongpoomchai R, et al. MRI contrast enhancement of liver pre-neoplasia using iron-tannic nanoparticles. RSC Advances. 2020;10(58):35419-25.
6. Saowalak K, Titipun T, Somchai T, Chalermchai P. Iron(III)-Tannic Molecular Nanoparticles Enhance Autophagy effect and T(1) MRI Contrast in Liver Cell Lines. Scientific reports. 2018;8(1):6647.
